# The effect of fatigue on electromechanical response times in basketball players with and without persistent low back pain

**DOI:** 10.1038/s41598-022-21940-8

**Published:** 2022-10-25

**Authors:** Sajjad Abdollahi, Rahman Sheikhhoseini, Mohammad Mohsen Roostayi, Wendy E. Huddleston

**Affiliations:** 1 Department of Corrective Exercise and Sport Injury, Faculty of Physical Education and Sport Sciences, Allameh Tabataba’i University, Western Azadi Sport Complex Boulevard, Hakim Highway, Tehran, Iran; 2 Department of Physiotherapy, School of Rehabilitation, Shahid Beheshti University of Medical Sciences, Tehran, Iran; 3 Department of Rehabilitation Sciences and Technology, College of Health Sciences, University of Wisconsin-Milwaukee, Milwaukee, Wisconsin USA

**Keywords:** Physiology, Health care

## Abstract

Typically, athletes alter movement mechanics in the presence of back pain, but the effect of these changes on lower extremity injury risk is not well understood. This study aimed to compare the effect of fatigue on electromechanical response times during a choice reaction task in basketball players with and without persistent low back pain. Twenty-four male basketball players participated. Total reaction time (TRT), premotor time (PMT), and electromechanical delay (EMD data were recorded before and after fatigue. The chronic low back pain (CLBP) group had significantly longer EMD in Med gastrocnemius (*p* = 0.001) and Tibialis anterior (*p* = 0.001), and shorter EMD in Vastus Lateralis (*p* = 0.001), Vastus Medialis Oblique (*p* = 0.003), and Semitendinosus (*p* = 0.025) muscles after fatigue. PMT in the CLBP group had longer than the Non-CLBP in Vastus Lateralis (*p* = 0.010), Vastus Medialis Oblique (*p* = 0.017), Semitendinosus (*p* = 0.002). Also, TRT was longer in knee flexion (*p* = 0.001) and ankle plantarflexion (*p* = 0.001) muscle groups. The different effects of fatigue on electromechanical response times of the knee and ankle in people with CLBP may represent the effect of an axial injury on lower extremity injury risk factors in situations of higher cognitive load, similar to competitive play.

## Introduction

The most frequent human musculoskeletal disorder is chronic low back pain (CLBP) that is identified by pain, limitation of hip range of motion^1^, reduced muscle strength of hip abductor/extensors and knee extensors^2^, muscular spasms, and postural disorders^3^. Clinicians tend to focus on the CLBP itself, however, CLBP might cause altered motor control of the lower extremities owing to pain-inhibitory mechanisms^4–6^. This change may cause altered or abnormal knee joint loads, which may be considered a central factor of non-contact anterior cruciate ligament (ACL) injury mechanisms^7,8^. Changed or abnormal lower extremity (LE) neuromuscular control during the performance of dynamic sports landings is increasingly suggested to contribute to ACL injury rates^7–10^. Dynamic muscular control of knee joint alignment^11^, specifically differences in muscle performance, firing patterns, and muscle strength, might be partly responsible for the incidence of ACL injury^12^. Additionally, knee joint abduction loads (e.g., occurring within landing tasks of dynamic sports) induce extreme ACL loads^13^. Given that lower extremity mechanics change in athletes with low back pain^14^, understanding the underlying neuromuscular factors related to dynamic knee loading during single-leg landing seems critical among this population and will provide critical insight into the potential for increased lower extremity injury risk.

Because of the spontaneous and dynamic nature of sports, it is critical to monitor any potential alterations in the timing of muscle activation caused by CLBP due to required quick and unexpected movement reactions^15–18^. Taking into consideration that athletes often react and produce such responses within a few hundred milliseconds^19^, the selected neuromuscular control of the desired movement may not succeed in accommodating the rapidly-changing external environment, increasing the possibility of injury^15,16,18^. For an assigned sport task or exercise, the selected movement is first controlled by the ability to react or respond to outside stimuli^19,20^. These reaction times are comprised of two acute phases, namely pre-motor time (PMT)^21^ and electromechanical delay (EMD)^21,22^, which are considered components of total reaction time (TRT)^21^. These temporal periods represent when the selected movement responses are initiated (PMT), the relative timing of the fundamental muscle activation strategy (EMD), and eventually, implementation success (TRT)^20,23^. Investigating these phases separately might further elucidate the specific timing for an increased potential for injury. For example, PMT changes during dynamic landings among athletes may influence the muscle's ability to stabilize the LE, especially the knee joint, against large external loads arising at the impact time of the foot contacts the floor^24^.

Additionally, individuals with CLBP experience alterations to the processes and complexity of motor control^14^. This causes these motor control deficit alterations to manifest as a spectrum of hypermobility to hypomobility in the affected segments, as well as minor changes in muscle coordination to complete avoidance of movement. Furthermore, neuromuscular control during functional activities is provided via feedforward (pre-activity), reflexive (short and long latency reflexes), and voluntary mechanisms^25^. Previous studies^24,26,27^ have shown that the frontal plane LE stability during landings may be predicted by the electromechanical response times of muscles that govern the resulting external knee loads. So, understanding relations between electromechanical response time after fatigue and resultant activity patterns and timing of muscle activity in athletes with CLBP thus represents another key focus of this paper.

Fatigue is an additional factor that may influence LE control^28^. Fatigue comes from physiological mechanisms at the central and peripheral levels^28,29^. Fatigue might affect afferent neuromuscular pathways, commonly perceived as the proprioceptive deficit^29,30^ and the efferent neuromuscular pathway, as evidenced through delayed muscle response^31^. Fatigue clearly may affect the force-producing capacity and the time specifications of the neuromuscular mechanism and delays in muscle responses as measured by electromechanical response time^32^. Analysis of temporal measures in conditions of fatigue, combined with LE mechanics changes associated with CLBP, may provide an even clearer picture of LE injury risk in this population.

The hip and lumbopelvic muscles play important roles in stabilizing the knee in basketball players by providing proximal LE stability, although these muscles may be also directly involved in CLBP. The purpose of this study, given the limited evidence base, was to compare the effect of fatigue on TRT, PMT, and EMD times of key knee and ankle muscle groups that would not be directly involved in CLBP during a choice reaction task (CRT) in basketball players with and without CLBP to determine the effect of CLBP on LE muscle activation patterns separate from the injury itself.

## Methods

### Subjects

For the current study to have 90% statistical power with an alpha level of 0.05, it would require at least 22 (same-sex) volunteers, according to data from a previous investigation on how neuromuscular fatigue impacts electromechanical delay (EMD) values. The power calculations were based on an effect size of 1.47 when investigating the impact of fatigue on EMD values for hip flexor muscles^33^. The inclusion criteria for athletes with CLBP and non-CLBP were: players ranging from 20 to 35 years old who have at least four years' history of playing in the Iranian super-league and first division league. Additionally, testing included subjects with nonspecific CLBP (≥ 12-week duration). A professional physical therapist with 15 years of experience treating musculoskeletal pain issues made the diagnosis of CLBP in the athlete (CLBP that pain exacerbate/alleviate with lumbopelvic movement, presence of no red flags, and not caused by a recognizable, known specific pathology). The exclusion criteria for CLBP patients were subjects with previous history of lumbar surgery or a medical condition with the contraindication of movement therapy, including acute (not re-occurring) low back injury occurring during the previous 12 weeks, presence of radiculopathy signs including radiating pain to the lower extremities, lower limb motor deficit and muscle weakness, and history of vertebral fractures. Prior to starting the investigation, study approval was obtained from the Biomedical Research Ethics Committee of Allameh Tabatab'i University (ATU) (Ethics code: IR.ATU.REC.1399.015), and all participants gave written informed consent. Authors confirm that all research was performed in accordance with relevant guidelines/regulations. All participants wore spandex bike shorts, sports shoes, and a sport brassier during testing.

### Experimental design

We recorded three-dimensional (3D) ground reaction force (GRF) during a dynamic choice reaction task (CRT) prior to and following exposure to a functional fatigue protocol. Prior to testing, participants had 10 min time to the general warm-up. The dominant leg was determined before data collection. The dominant leg was found by having a patient drop from a step onto a single limb for 3 times. The leg used for landing minimally for 2 trials was considered a dominant leg^17^.

**Figure 1:**
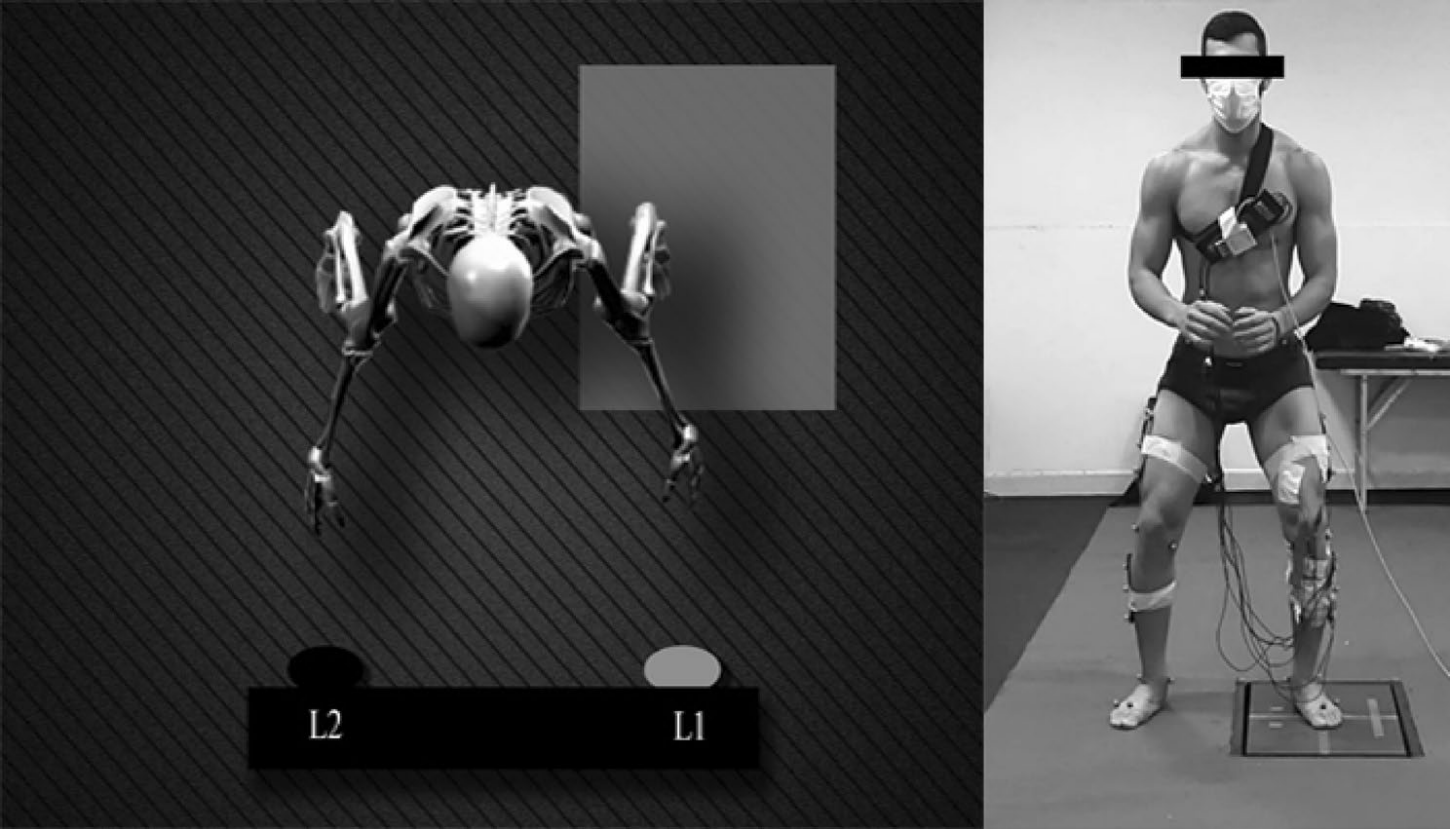
Participants were needed to respond as rapidly as possible to randomly ordered light stimuli for the choice reaction time task. By turning on L1 light, subjects performed a bilateral vertical, landing on the left foot and then jumping as rapidly as possible to the right. The opposite sequence was cued by the L2 light.

**Figure 2:**
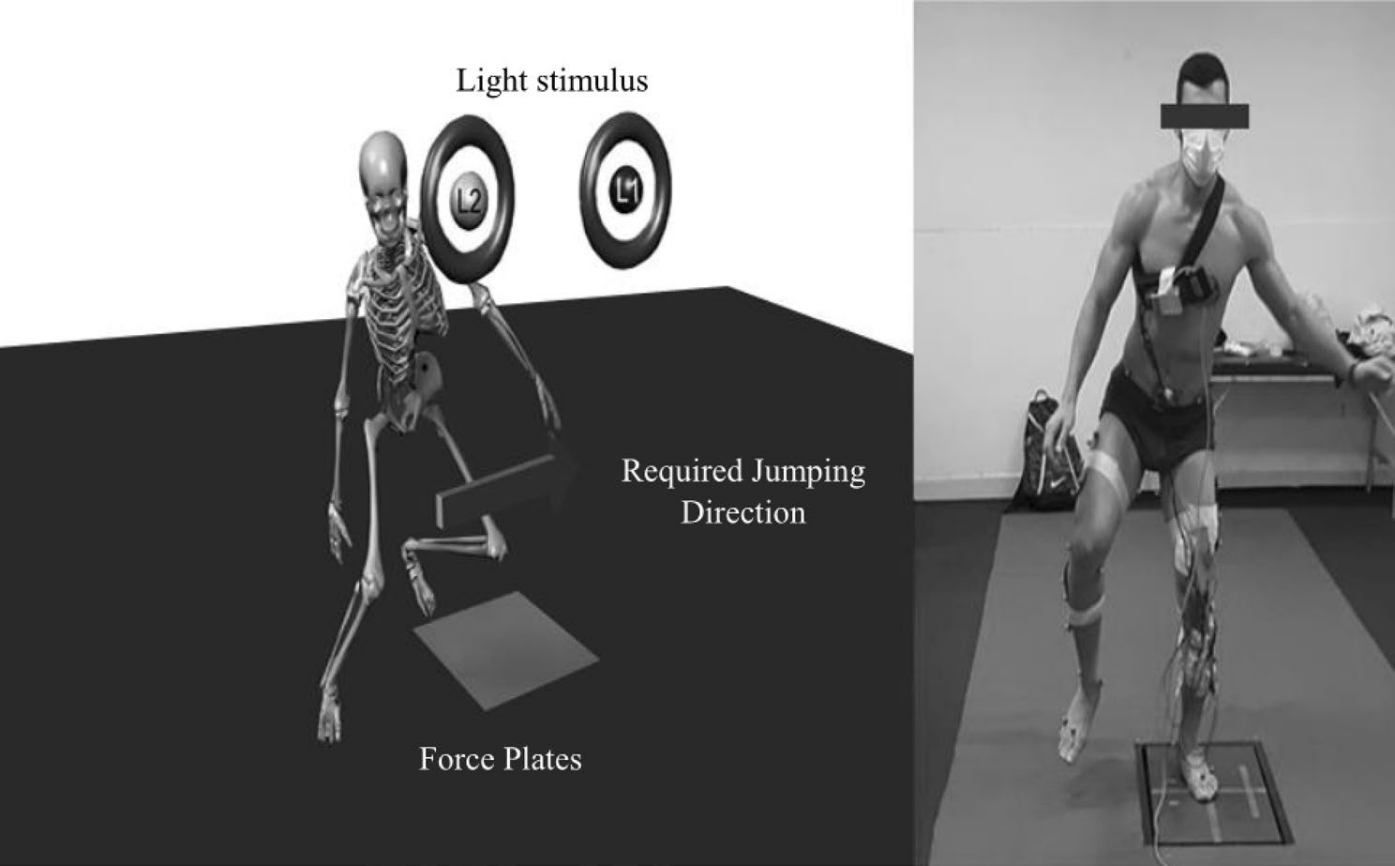
Subjects reacted to a random light stimulus and moved in the appropriate direction (right (L1) or left (L2) upon landing on the cued limb.

All participants performed CRTs both pre-fatigue and post-fatigue. Specifically, subjects were required to execute one of two unanticipated landings which were ordered in a random way, and cued by activating a light (L1, or L2) before the landing period. Participants faced two lights spaced 30 cm apart and 1 m high in front of them at waist height while standing with their feet 50 cm apart from the center of the force plate (Fig. 1). The motor task and light placements were coupled to reflect unexpected movements that frequently arise during gameplay^34^. The L1 light cue indicated the participant should perform a vertical jump originating from both feet and then landing on the left foot. Upon landing, the participant then instantly and aggressively pushed off laterally and landed on the right foot (Fig. 2). Illumination of L2 indicated the participant should perform the above sequence landing first on the right foot, pushing to the left and subsequently landing on the left foot.

Each single-leg landing trial was started by random illumination of either L1 or L2. For the study trials, the L1 and L2 stimuli were automatically triggered via a light beam switch. For the trials, participants knew the landing pattern prior to the initial vertical jump. Each subject was thus required to perform a minimum of 3 pre-fatigue and post-fatigue unanticipated landing trials to ensure adequate data were available for each pre-fatigue and post-fatigue jump condition. Each participant was instructed to complete three trials of both anticipated and unanticipated landings before testing began to familiarize him with the study protocol. Only the data from trials in which the participant's dominant leg touched the force plate were evaluated. Testing continued until three trials for the dominant leg were completed from the random sequence of lights.

The data were obtained and recorded throughout synchronous ground reaction force (GRF) (1200 Hz) and lower limb muscle electromyography (EMG; 2400 Hz). Muscle EMG measurement was performed only for the dominant limb for four muscles using an eight-channel analog system (Megawin, Germany). For each electrode site, the skin was first shaved and cleaned using alcohol. Pregelled Ag/AgCl bipolar surface EMG electrodes with a diameter of 1.1 cm and inter-electrode distance of 3.5 cm were put down over the muscle bellies and in line with the muscle fibers of the Vastus lateralis (approximately at 2/3 on the line from the anterior superior iliac spine to the lateral side of the patella), Vastus medialis oblique (approximately at 80% on the line between the anterior superior iliac spine and the joint space in front of the anterior border of the medial ligament), Biceps femoris (approximately at 50% on the line between the ischial tuberosity and the lateral epicondyle of the tibia), Semitendinosus (approximately at 50% on the line between the ischial tuberosity and the medial epicondyle of the tibia), med gastrocnemius (approximately on the most prominent bulge of the muscle), lateral gastrocnemius (approximately at 1/3 of the line between the head of the fibula and the heel), and tibialis anterior (approximately at 1/3 on the line between the tip of the fibula and the tip of the medial malleolus) of each limb based on established procedures^35^. Moreover, a single ground electrode was placed on the medial femoral condyle of the dominant limb. Electrodes were connected to the skin by adhesive stickers and then wrapped with 1.5-inch elastic bands.

**Figure 3:**
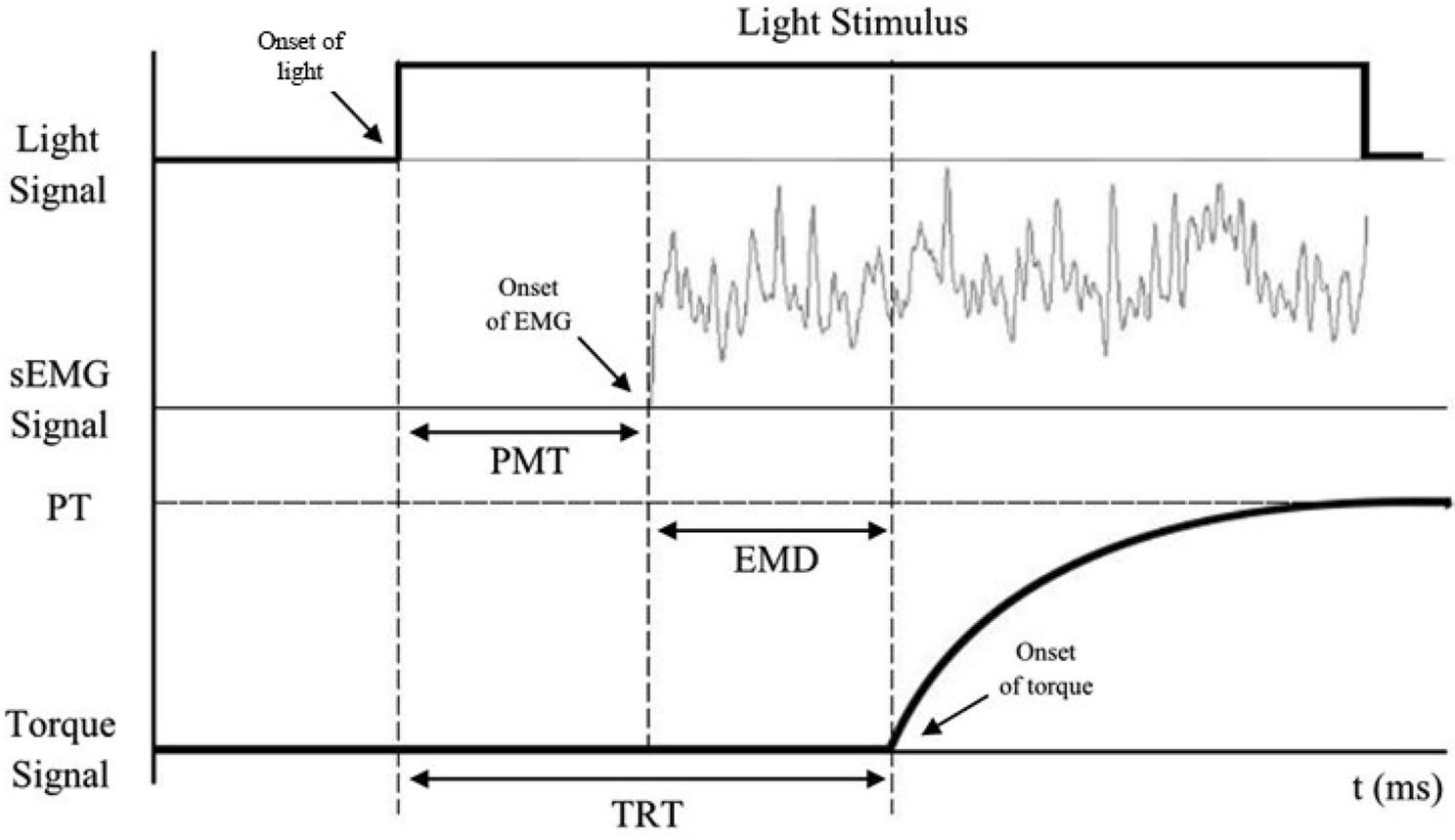
Schematic representation of TRT determination during an unanticipated trial. TRT—total reaction time, PMT—premotor time, EMD—electromechanical delay, PT—peak torque.

The muscle EMG data were changed to a linear envelope using full-wave rectification and low-pass filtered by a second-order, phase-corrected Butterworth filter with a cut-off frequency of 20 Hz to reduce motion artifacts^36^. The onset of muscle activation for the dominant limb was computed during the unanticipated performance, described as the time in which the signal was greater than three times the activity obtained during rest for the minimum of 50 ms^36^. The levels of activity at rest was based on EMG data collected during 5 s of stationary standing prior to initiating the jumps. Execution time was also determined for each trial, being the time between the initial stimulus onset and the initiation of the movement response, denoted as the first instance that the vertical GRF in the push-off limb rose above 10 N (Fig. 3).

**Figure 4:**
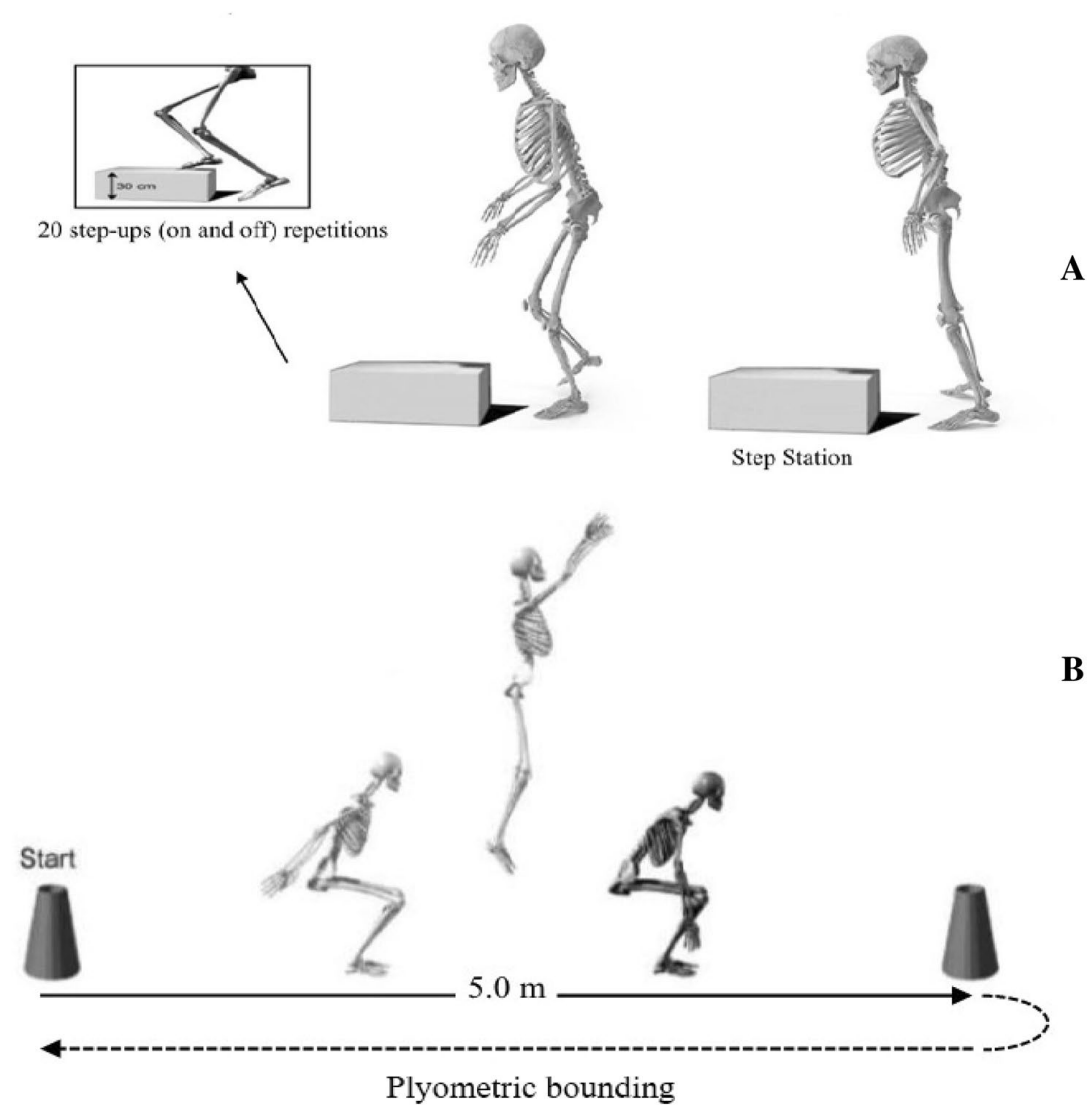
Subject cyclically performed a series of step-up (**A**) and bounding (**B**) tasks at maximum intensity for a 4- min period as a means to induce general fatigue.

Following the pre-fatigue anticipated and unanticipated trials, subjects performed the fatigue protocol for 4 min. This protocol included a group of drills without interruption that loosely mimicked tasks closely associated with actual gameplay and the same as general fatigue patterns used formerly^29,37^. Specifically, participants first executed 20 step-up and step-down motions as fast as possible onto/off a 20 cm step. Immediately afterward, the participants executed a group of plyometric bounding motions without interruption for a distance of 5.0 m, rotated 180°, and finished the second group of bounds back to the starting point (Fig. 4 A and B). The participants were trained to land and move into a deep knee-flexion position for each bounding motion as the body was quickly slowed down. Following getting to their maximum flexion position, the subjects rapidly and instantly rebounded into the subsequent jump stage, the same as a plyometric bounding task. The whole sequence of the two activities was repeated as many times as possible during the 4-min time.

The heart rate of every participant was constantly monitored throughout the fatigue protocol and recorded using a Polar heart rate monitor attached around the chest (S520; Polar Electro Inc., Lake Success, NY, USA) to obtain a general subject-specific standard of fatigue. Heart rate data were downloaded to a laptop computer on completion of all testing, from which the maximum heart rate attained throughout the protocol was calculated. The intensity of the fatigue protocol was measured by Rated Perceived Exertion (RPE) while the participants performed the fatigue protocol and immediately at completion. Participants were considered fatigued when their RPE was reported to be at least 6/10. The fatigue protocol was repeated until the patient reached the target intensity if they reported an RPE score below 6.

### Biomechanical analyses

Kinetics were gathered at the time of CRTs using a force plate (Bertec Corp., Columbus, Ohio) sampling at 1200 Hz. The data of the dominant limb were gathered. All kinetic data were utilized to extract the study desired variables. The desired variables' mean average values across three trials were taken. An unanticipated performance was regarded as successful when the individuals landed on the force platform during CRT, where it was necessary to land and balance on the lower limb for a minimum of 1 s prior to the lateral push off (Fig. 2). The kinetic data of landing performances were time normalized to 100% of posture, with initial contact and toe-off described as the time the vertical GRF was first over and under 10 N, respectively^18^. The CRTs were time-normalized (100%) between vertical GRF data points apparent during the push-off stage of the motion. A biomechanics professional wrote the MATLAB software code used to analyze the biomechanical data. Missing data shorter than a duration of 20 frames, which happened seldom, were interpolated using standard techniques.

### Statistical analysis

The total reaction times (TRT), pre-motor times (PMT), and electromechanical delay (EMD) from three trials were averaged, for each subject and each trial type, and used in data analyses. The Shapiro–Wilk test of normality examined data distribution. Data were analyzed using ANCOVA by considering the pretest measures as covariate variables. The significance level was set as 0.05 for all the tests. All analyses were performed using SPSS software version 24.0 (SPSS, Chicago, IL, USA).

### Ethics approval and consent to participate

Prior to starting the investigation, study approval was obtained from the Biomedical Research Ethics Committee of Allameh Tabatab'i University (ATU) (Ethics code: IR.ATU.REC.1399.015), and all participants gave written informed consent. Authors confirm that all research was performed in accordance with relevant guidelines/regulations. Informed consent from all subjects were given for publication of identifying information/images in an online open-access publication.

## Results

Demographic data for the participants are shown in Table 1. No significant differences were found in age, height, body mass, BMI, PHR, and RPE among the two groups.

**Table 1:** Participant demographics.

Variable	CLBP (N = 12)	Non-CLBP (N = 12)	*P* value
	Mean ± SD	Mean ± SD	
Age (years)	23.8 ± 1.8	23.5 ± 2.2	0.241
height(cm)	183.9 ± 6.7	188.0 ± 7.8	0.264
Body Mass (kg)	79.6 ± 8.9	87.0 ± 10.2	0.227
BMI (kg.m2 )	23.4 ± 1.3	24.5 ± 1.1	0.498
PHR (bpm)	139.8 ± 1.9	142.9 ± 1.0	0.134
Intensity of fatigue (RPEs)	7.2 ± 0.6	6.8 ± 0.5	0.103
VAS (mm)	7.9 ± 0.6	N/A	–
ODI (pts)	22.9 ± 11.9	N/A	–

*BMI* Body Mass Index, *PHR* Peak Heart Rate, *RPEs* Rate of Perceived Exertion scale, *VAS* Visual Analogue Scale of current pain, *ODI* Oswestry Disability Index.

The total reaction time (TRT) data are provided for each muscle group (knee flexion/extension and ankle dorsiflexion/plantarflexion) between the CLBP and non-CLBP groups before and after fatigue (Table 2). A significant main effect of fatigue for knee flexion (*p* = 0.001) and ankle plantarflexion (*p* = 0.001) were found for TRT. Moreover, after fatigue, the CLBP group had a longer TRT than the non-CLBP in the ankle plantar and knee flexion muscle groups (*p* < 0.001).

**Table 2:** Means and standard deviations of total reaction time, premotor time, and electromechanical delay for muscle groups/muscles in CLBP and Non-CLBP groups for the unanticipated trials.

Variable	CLBP (N = 12)		Non-CLBP (N = 12)		*P* value^†^
	Non-Fatigue	Fatigue	Non-Fatigue	Fatigue	
	Mean ± SD	Mean ± SD	Mean ± SD	Mean ± SD	
TRT (ms)					
Knee extension	19.37 ± 9.35	20.79 ± 1.17	18.19 ± 3.01	19.25 ± 3.02	0.089
Knee flexion	22.52 ± 5.56	23.50 ± 2.45	16.44 ± 3.14	18.24 ± 1.62	< 0.001**
Ankle dorsiflexion	11.21 ± 6.78	10.42 ± 1.23	12.76 ± 3.48	14.59 ± 2.97	0.070
Ankle plantarflexion	7.87 ± 6.94	12.27 ± 2.57	14.46 ± 3.66	16.48 ± 3.39	< 0.001**
PMT (ms)					
VL	3.91 ± 1.15	7.50 ± 1.03	3.60 ± 2.62	5.51 ± 2.18	< 0.010**
VMO	5.43 ± 2.25	7.13 ± 1.41	2.90 ± 2.15	4.68 ± 2.87	< 0.017**
BF	5.65 ± 2.37	4.08 ± 2.18	5.25 ± 2.66	4.31 ± 2.69	0.239
SM	5.98 ± 2.78	8.23 ± 1.20	4.77 ± 2.37	5.93 ± 2.24	< 0.002**
MG	5.85 ± 8.86	− 4.20 ± 4.03	− 11.65 ± 3.03	− 3.09 ± 3.00	< 0.038**
LG	3.75 ± 9.09	6.07 ± 4.88	− 3.34 ± 3.51	− 5.84 ± 3.09	0.272
TA	− 3.60 ± 6.11	− 5.17 ± 4.39	− 2.89 ± 3.14	− 2.89 ± 3.28	0.239

*TRT* total reaction time, *PMT* pre-motor time, *VL* vastus lateralis, *VMO* vastus medialis oblique, *BF* biceps femoris, *SM* semitendinosus, *MG* med gastrocnemius, *LG* lateral gastrocnemius, *TA* tibialis anterior, *SD* standard deviation.

**P value < 0.05 is considered to be statistically significant.

^†^Statistically significant differences between the CLBP and non-CLBP groups.

**Figure 5:**
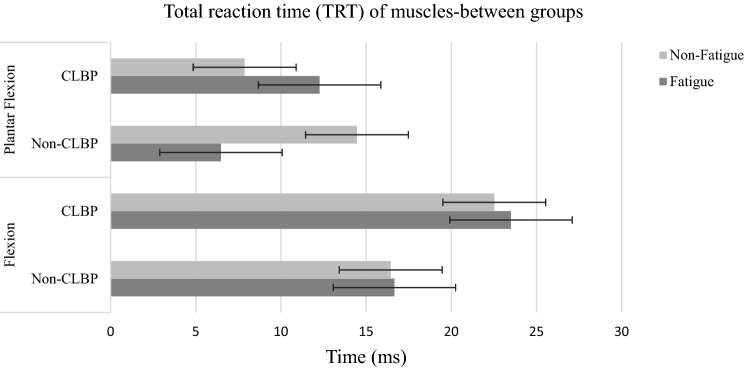
TRT for each muscle by groups (CLBP and Non-CLBP) in non-fatigued and fatigued. Flexion: Knee flexion, Plantar Flexion: Ankle Plantar Flexion.

**Figure 6:**
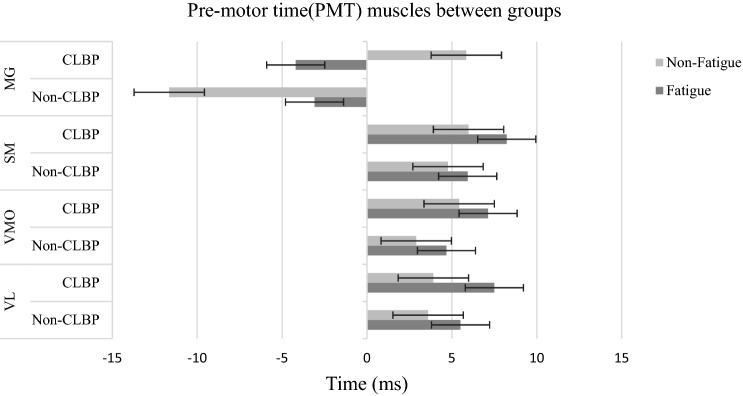
PMT for each muscle by groups (CLBP and Non-CLBP) in non-fatigued and fatigued. VL, Vastus Lateralis; VMO, Vastus Medialis Oblique; SM, Semitendinosus; MG, Med gastrocnemius.

When evaluating the premotor time by muscle, a statistically significant effect of fatigue was found in Vastus Lateralis (VL) (*p* = 0.010), Vastus Medialis Oblique (VMO) (*p* = 0.017), Semitendinosus (SM) (*p* = 0.002), and medial Gastrocnemius (MG) (*p* = 0.038). The CLBP group had a longer per-motor time (PMT) than the non-CLBP in all of above-mentioned muscles (Figs. 5, 6).

**Figure 7:**
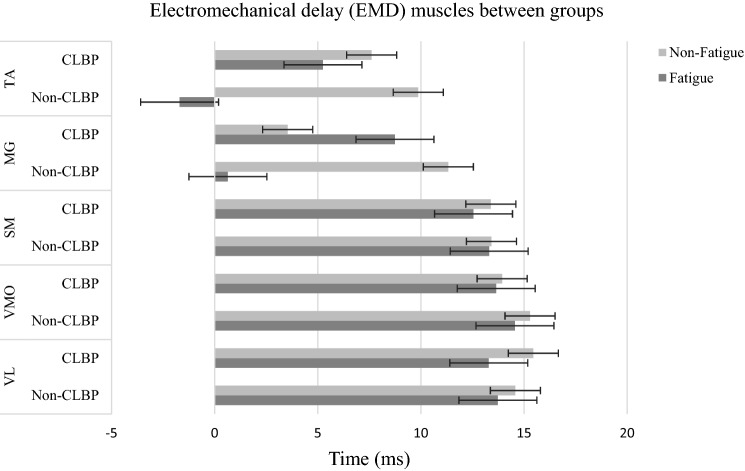
EMD for each muscle by groups (CLBP and Non-CLBP) in non-fatigued and fatigued. VL, Vastus Lateralis; VMO, Vastus Medialis Oblique; SM, Semitendinosus; MG, Med gastrocnemius; TA, Tibialis anterior.

A significant main effect of fatigue existed for Vastus Lateralis (*p* = 0.001), Vastus Medialis Oblique (*p* = 0.003), Semitendinosus (*p* = 0.025), medial Gastrocnemius (*p* = 0.001), and Tibialis anterior (*p* = 0.001) muscles on electromechanical delay (Table 3). The CLBP group had longer EMD than the Non-CLBP group in Med gastrocnemius and Tibialis anterior muscles (*P* < 0.001). But, the Non-CLBP group has longer EMD than the CLBP in the Vastus Lateralis (*P* < 0.001), Vastus Medialis Oblique (*p* = 0.003), and Semitendinosus (*p* = 0.025) after fatigue (Fig. 7).

**Table 3:** Means and standard deviations of electromechanical delay for muscle groups/ muscles in CLBP and Non-CLBP groups.

Variable	CLBP (N = 12)		Non-CLBP (N = 12)		*P* value^†^
	Non-Fatigue	Fatigue	Non-Fatigue	Fatigue	
	Mean ± SD	Mean ± SD	Mean ± SD	Mean ± SD	
EMD (ms)					
VL	15.45 ± 9.57	13.29 ± 1.65	14.58 ± 4.83	13.73 ± 4.14	< 0.001**
VMO	13.94 ± 10.82	13.65 ± 1.93	15.29 ± 3.98	14.56 ± 4.70	< 0.003**
BF	13.71 ± 10.09	16.70 ± 2.58	12.94 ± 3.05	12.93 ± 4.24	0.578
SM	13.39 ± 9.25	12.55 ± 1.72	13.42 ± 4.73	13.31 ± 4.12	< 0.025**
MG	3.54 ± 6.00	8.74 ± 4.87	11.33 ± 1.19	0.64 ± 2.07	< 0.001**
LG	4.79 ± 6.87	5.53 ± 5.06	2.60 ± 2.28	3.39 ± 1.73	0.054
TA	7.61 ± 2.43	5.25 ± 4.34	9.87 ± 2.05	− 1.70 ± 1.11	< 0.001**

*EMD* electromechanical delay, *VL* Vastus Lateralis, *VMO* Vastus Medialis Oblique, *BF* Biceps Femoris, *SM* Semitendinosus, *MG* Med gastrocnemius, *LG* Lateral gastrocnemius, *TA* Tibialis anterior, *SD* Standard deviation.

**P value < 0.05 considered to be statistically significant.

^†^Statistically significant differences between the CLBP and Non-CLBP groups.

## Discussion

Our primary finding was that the CLBP group has a longer TRT than the non-CLBP group in the ankle plantar flexion and knee flexion muscle groups after fatigue. Also, for VL, VMO, SM, and MG muscles in the CLBP group had longer PMT than the non-CLBP in all of these muscles after fatigue.

In athletes with CLBP, longer PMT and TRT may be related to compromised spinal and/or supraspinal pathways due to injury that may affect timing to unexpected events^8,17^. If so, these results may provide insight into potentially injurious loading strategies during maneuvers^38^. CLBP, through timing limitations and delay in muscle activation, may cause strategic changes in the transfer of load through the pelvic girdle to the lower limb and increase the risk of lower limb injuries among athletes^39,40^. This conclusion assumes TRT and PMT are predictors of lower limb injuries, especially non-contact ACL injury risk^41^. In summary, the current results support the possible higher risk of ACL injuries in people with CLBP during fatigue.

To our knowledge, no previous study has been performed on lower limb muscle TRT and PMT measurements for landing performances in individuals with CLBP. However, PMT values have been shown to be significantly more prolonged than values reported for other explosive motion tasks in healthy, asymptomatic individuals (e.g., sprinting)^42^. The PMTs of lower limb muscle are constrained by task complexity^43^, integrated upper and lower limbs coordination needs^44^, and skill/experience level^23^. The PMT measurements are different among muscle groups, probably indicating correspondingly diverse contributions to the motions required by the task. Specifically, in our choice reaction task, it is necessary for participants to extend at the hip, knee, and ankle joints while pushing off quickly. Therefore, the usual activation of the hip and knee extensor muscles before the antagonistic flexor groups is intuitive. Short PMTs of the Vastus medialis oblique and Vastus lateralis are more difficult to explain. However, shorter PMTs might help in the stabilization of the knee joint during the push-off period after a bout of fatigue^11,18,45–47^. The rather short PMT for the bi-articular lateral hamstring might have been obvious for similar reasons at the knee or may help in initial hip extension.

Our other finding was that PMT was somewhat diminished in the non-CLBP group yet significantly increased in the CLBP group following a bout of fatiguing exercise. PMT measures for the dynamic knee stabilizers, particularly VMO and medial hamstrings, were longer. In people who have CLBP, lower limb muscle PMTs effectively shows how long the muscles were active before initial external load application^24,47^. So, considering abduction loads increased rapidly after fatigue for both groups, PMT magnitudes will directly impact the successful support of these loads^38,48^. Therefore, although it is less intuitive, it is likely that the increase in the externally exerted abduction load, needing a greater supporting muscle force, results in increasing muscle PMT, proposed to correspond to the extent of force output^49^. Consequently, the conclusions of the present study should be taken into consideration bearing this major fact in mind.

The time delay from the earliest onset of EMG activity to the initial onset of force generation is considered the definition of EMD^21,22,46^. The CLBP group had longer EMD than the Non-CLBP group in MG and TA muscles, but the Non-CLBP group had longer EMD than CLBP in the VL, VMO, and SM following a bout of fatiguing exercise. Here, the results of our studies are consistent with previous studies. Zhou et al.^50^ reported a significant increase in EMD for the rectus femoris muscle (from 40.4 to 63.4 ms) due to fatigue.

In combination, PMT and EMD determine the absolute shortest time possible to produce muscle tension (i.e., TRT) and potential response to knee joint perturbations. Factors that lengthen each time component delay the development of muscular tension, compromising dynamic joint stabilization and increasing the possibility of injuries^8,17,34,51^. Lengthening of any component of the TRT may be due to alteration and adaptation the nervous system of athletes with CLBP, which transfers to the joint, and eventually leading to riskier biomechanics^14,52–55^. So, the results of the present study suggest there may be a greater potential in the non-CLBP group to compensate for possible contraction force failure and delays in comparison to the CLBP group under the same levels of fatiguing exercise.

Our results suggested that people with CLBP have longer PMT and EMD in medial hamstrings, vastus lateralis, and VMO, so there is a possibility for CLBP individuals to be at an increased risk of ACL injury during dynamic landings through an external knee abduction loading mechanism. Precisely, longer PMT of these muscles during such tasks might not give sufficient time for the muscles to stabilize the joints in individuals with CLBP. Fatigue in healthy individuals results in a significantly stronger reliance on neuromechanical signals from the LEs for controlling posture during maneuvers^56^. On the contrary, neuromechanical signals may deteriorate due to muscle fatigue, which leads to inaccurate signals about LE proprioception and kinesthesia in athletes with CLBP^40,56^. As a result, our results can be explained by the negative influence of fatigue on the muscle receptors and neural pathways, and thereby on neuromechanical signals like proprioception in athletes with CLBP.

Our findings suggest that athletes with CLBP may be more prone to other injuries after becoming fatigued. Therefore, it can be suggested that athletes with CLBP add training techniques to their regimens to avoid being overly exhausted when participating in physical activity or sporting events. In order to successfully counteract the potentially risky biomechanical consequences of fatigue in people with CLBP, we can advise athletes with CLBP to pay more attention to proprioceptive and motor control training to be incorporated into an ACL injury prevention approach.

## Limitations

Our evaluations were conducted in a laboratory setting, so some caution is warranted when generalizing our results to the actual sports fields. Moreover, the study's cross-sectional nature prevents understanding the real possible relationship between the study variables and lower extremity injuries. Also, this study examined only landing maneuvers, so our results may not be generalized to other sports or other tasks. Future research is required to determine which variable may be more determinant or relevant for athletes' performance hazards because the potential relationship between the study factors and sport performance remained unclear.

## Conclusion

The results showed that fatigue might negatively affect TRT, PMT, and EMD more in athletes with CLBP than those without. The effect of fatigue on specific muscle TRT, PMT, and EMD measures in people with CLBP may represent a worst-case scenario, such as increased anterior cruciate ligament injury risk, during a choice reaction task.

## Supplementary Information

Supplementary Information.

## Data Availability

The raw data and material will be available online after publishing the paper as a supplementary file in the journal.
